# Supplementary material for: Deep learning-based no-reference image quality assessment framework for Cryptosporidium spp. and Giardia spp
Source: PLoS One. 2026 Jan 20;21(1):e0341160. doi: 10.1371/journal.pone.0341160 (PMC12818675; doi:10.1371/journal.pone.0341160)
Supplement: Attachment — Submitted filename: Response to reviewers.docx [file pone.0341160.s002.docx]

15 August 2025

Dear Editor and Reviewers,

Thank you very much for considering our manuscript entitled “**Deep Learning-Based No-Reference Image Quality Assessment Framework for Cryptosporidium spp. and Giardia spp.**”. We really appreciate your valuable suggestions and comments which help us to enhance the quality of our paper. We have revised the manuscript accordingly, with details below:

*Reviewers #1 comments:*

1. *Figures are missing in the main manuscript.*

- According to the PLOS ONE submission policy (https://journals.plos.org/plosone/s/submission-guidelines), figures were submitted as separate files and were not embedded in the main manuscript.

2. *The authors should more explicitly emphasize the novelty of addressing NR-IQA in parasitic microscopy images, especially in the introduction and conclusion, to better contextualize their contribution to public health diagnostics.*

- The manuscript was revised especially on **Introduction (lines 152 – 157)** and **Conclusion (lines 591 – 594)** to more explicitly emphasize the novelty and significance of our work. The revisions now clearly state that this study presents the first dedicated NR-IQA framework for parasitic microscopy images, focusing on *Cryptosporidium* and *Giardia*, which are of significant public health concern. We have strengthened the motivation by highlighting the critical role of image quality in reliable parasite detection and the absence of NR-IQA solutions in this domain. These changes better contextualize our contribution within the scope of public health diagnostics, particularly in resource-limited settings.

3. *While the methodology is well-structured, the authors should consider summarizing repetitive elements (e.g., similar pipeline diagrams) and improving flow between sections to enhance readability.*

- Revised the **Proposed Framework section (lines 326 – 333)** to reduce redundancy and improve readability. Only included the pipeline diagram of the PRIQA framework (Fig. 4) and removed the separate pipeline diagrams for PEIQA and PDIQA. The pipelines for PEIQA and PDIQA, which follow the same structure as PRIQA with different CNN backbones, are now explained concisely in the text.

4. *The training set includes only 23 original images with synthetic distortions, which is small for training deep models. Real-world generalization may be compromised.*

- To address the limitation of using a small dataset of only 23 original images, we have adopted several strategies that are widely recognised for enhancing model performance and generalisation in data-scarce scenarios:

Data Augmentation & Synthetic Distortions: We generated diverse synthetic distortions to mimic real-world degradation observed in parasitic microscopy. This is not only increased the training set size but also helped the model learn robust features. In addition to that, all the networks were pre-trained with ImageNet datasets (**line number 247 – 252)**. According to (<https://doi.org/10.59720/20-130>), a small dataset is sufficient to train pre-trained models.

- Acknowledged the data limitation and note that future work will focus on expanding the dataset with real-world samples to further validate the model’s robustness in practical deployment **(refer line 610 – 618)**.

5. *The paper lacks an ablation study analyzing the effect of each component (e.g., CNN backbone, regression model) on performance.*

- Revised manuscript includes an ablation study shown in **Table 9** on feature extraction layers across different CNN backbones (ResNet-101, EfficientNet-B0, and DarkNet-53) in **“Analysis of Ablation” section (lines 487 – 509).** This analysis shows the effect of selecting different intermediate layers on performance (PLCC, SROCC, KROCC and RMSE). The findings suggest that deeper layer capture more abstract and semantically rich features that align more closely with human perception of image quality. Therefore, the deepest and best-performing layer from each of the top three CNNs was selected as the final feature representation for the PRIQA, PEIQA, and PDIQA models.
- For the regression models, we evaluated multiple options during the main experiments (e.g., Linear, Cubic SVM, Gaussian SVM, Tree-based models) and reported their comparative results in **Table 7.** To ensure fairness and reduce redundancy, the ablation study focused on feature extraction layers only, while the regression models were fixed to the best-performing ones identified in **Table 7.** This combination provides insight into how each component contributes to the overall performance of the proposed PRIQA framework.

6. *The distortion types are predefined and artificially induced. Natural distortions often found in field microscopy images should also be considered for broader applicability.*

- Although our current distortion types (Gaussian noise, blur and compression artefacts) are synthetically generated to stimulate common quality degradations. However, these artefacts occur on the microscopy. Distorted microscopic datasets are unavailable especially in Cryptosporidium spp. and Giardia spp. as this area remain unexplored yet. Added as future work to include real-world distortions. **Refer line 613 – 618.**
- In the conclusion section, the need to integrate real-world microscopy data affected by environmental noise, focus misalignment, non-uniform staining, and illumination variation is highlighted which is common in field diagnostics. **Refer line 615 – 618.**

7. *While MOS is a standard, relying solely on subjective scores without incorporating image-specific perceptual or structural quality metrics may limit robustness.*
   - Added a new section titled **“Correlation between MOS and FR-IQA Metrics”** **(line 429)**. Benchmarked the MOS with a well establish Full-Reference Image Quality Assessment (FR-IQA) namely Structural Similarity Index Metrics (SSIM), Multiscale SSIM (MS-SSIM), Feature Similarity (FSIM), and Information Weighted SSIM (IW-SSIM). Based on the comparison, we found that our MOS has recorded more than 0.68 against 4 FR-IQA metrics. According to (<https://doi.org/10.1016/j.mri.2016.03.006>.), correlation coefficients more than 0.68 shows that MOS & FR-IQA metrics are close. Refer **line 432 – 434.**
8. *Authors should add the time and space complexity of the proposed model to show the scalability and efficiency of the proposed model compared to the baseline models.*
   - Time and space complexity of the proposed model is added in **Table 7** in the revised manuscript. **Refer line 459.**
     i. *Prediction speed (obs/sec)* and *training time (sec)*, which reflect time complexity, and
     ii. *Model size (bytes)*, which reflects space complexity.
   - These metrics are reported for all evaluated CNN architectures thereby demonstrating the scalability and efficiency of the proposed PRIQA framework relative to baseline models.
   - Based on the findings in **Tables 7 and 8**, although ResNet-101 achieved the highest accuracy, its prediction speed was moderate (291.36 obs/sec) compared to faster models like ResNet-18 (2544.03 obs/sec) and GoogleNet (1514.10 obs/sec). EfficientNet-B0 and DarkNet-53 offered a balanced trade-off, combining high accuracy with faster prediction speeds and shorter training times. Model size is also important for memory-limited hardware. AlexNet, despite its large size (21.0 MB) and slow prediction speed (36.94 obs/sec), performed less competitively in terms of accuracy. These results suggest that while ResNet-101 is ideal for accuracy-critical tasks, EfficientNet-B0 and DarkNet-53 may be more practical choices for real-time or resource-constrained deployments. This is mentioned in **line number 474 – 484.**
9. *How sensitive is the model to the selection of hyperparameters? Perform some sensitivity analyses.*
   - Added a comprehensive sensitivity analysis from **line 511 - 541** to assess how hyperparameter choices affect model performance. For the top three CNN–regressor pairs (PRIQA: ResNet‑101 + Cubic SVM; PDIQA: DarkNet‑53 + Cubic SVM; PEIQA: EfficientNet‑B0 + Quadratic SVM), we systematically varied three SVM hyperparameters (epsilon, box constraint, and kernel scale) by ±25% and ±50% around the baseline.
   - The results, presented in Sensitivity Analysis section and illustrated in **Fig. 6** (line graphs), show that performance is generally robust to moderate hyperparameter changes. Only minor fluctuations in RMSE were observed across most settings, with EfficientNet‑B0 being slightly more sensitive to box‑constraint variations. These findings support that the selected hyperparameters strike a good balance between stability and accuracy, which is why the baseline "auto" settings were retained for the main experiments.
10. *The performance metrics demonstrate the efficacy of the framework, but more advanced statistical tests, such as Nemenyi and Wilcoxon tests, would improve the robustness of the results. These tests can provide further validation, especially in comparing classification accuracy.*
    - Added additional statistical significance tests from **line 560 - 588** to further validate our results. Specifically:
      i. Wilcoxon signed-rank test to compare the PRIQA framework with each baseline NR-IQA method across the 3 evaluation metrics (PLCC, SROCC, RMSE).
      ii. Nemenyi post-hoc test following the Friedman test to assess whether the observed differences among all compared models were statistically significant.
    - The detailed results are included in Statistical Significance Analysis from **line 560 - 588** and summarized in **Table 11**. These tests confirm that the performance improvements achieved by PRIQA over baseline methods are statistically significant at the 95% confidence level (α = 0.05), further strengthening the robustness of our findings.
11. *Code availability is crucial for reproducibility, and the authors should release the implementation code to ensure other researchers can replicate their findings. This is especially important for a field where transparency and reproducibility are vital.*
    - We have made the full implementation code publicly available. The repository includes scripts for feature extraction, distortion generation, and regression-based quality assessment, as well as pretrained CNN feature sets to facilitate replication. The code is accessible at: <https://github.com/Amirul-777/PRIQA---Parasite-ResNet-101-Image-Quality-Assessment-Study>. **Refer line 631 – 634.**
12. *The linguistic quality of the paper needs improvement, particularly in maintaining flow between sections and subsections by writing something in between the sections and subsections. The authors should ensure that transition sentences are added to improve readability.*
    - Manuscript has been revised to improve the overall flow and readability.
13. *Lastly, the conclusion should discuss potential limitations of the proposed model and outline future work directions.*
    - Conclusion section has been revised to explicitly discuss the limitations **(lines 610 – 625)** of the proposed PRIQA framework and outline directions for future work. The revised version now highlights that the dataset used in this study contained only synthetic distortions and that the generalizability of the model to naturally distorted or clinically acquired images remains to be tested. Noted that the regression models employed, although effective for the current dataset, may face scalability challenges with larger and more heterogeneous datasets. Finally, we outline potential future work directions, including exploring end-to-end deep learning approaches, testing on broader datasets, incorporating real-time feedback mechanisms, and developing lightweight models suitable for resource-constrained diagnostic settings. These additions, found in the revised Conclusion section, directly address the reviewer’s recommendation and strengthen the discussion of limitations and future research opportunities.

*Reviewers #2 comments:*

1. *With the size and composition of dataset, please specify the range of distortions, imaging conditions which will be required to assess the dataset and the strength of the model.*
   - The range of distortions and imaging conditions is already described in the Training and testing dataset subsection **(lines 178–208)** and summarized in **Table 1**. This section specifies the four types of distortions (Gaussian White Noise, Salt and Pepper Noise, Speckle Noise, and JPEG Compression) and their respective intensity levels (e.g., Gaussian noise σ = 10–90, JPEG quality factor = 10–50), as well as the imaging conditions used to acquire the reference images (microscope type, magnification, and illumination). The imaging conditions after applying the distortions were shown in **Fig. 2**. These details provide a comprehensive view of the dataset composition and support the robustness and generalizability of the proposed PRIQA model.
2. *The authors are suggested to review and refer some latest studies in this area (mention the work done and consider the studies of 2023, 2024).*

We have updated the **Introduction** **(lines 130–148)** to include several recent studies from 2023–2025 relevant to NR-IQA and biomedical image quality assessment. These additions discuss the latest works and provide a stronger context for the novelty of our proposed PRIQA framework.

3. *Add more detail about the regression model. Specify the types of regression models applied, their procedures and justification of using them in your study.*
   - Added description of the types of regression models, their functions, and rationale in **lines 290–295** and summarized in **Table 3**. This range of models was selected to cover both linear and nonlinear relationships and to compare parametric and nonparametric approaches. The procedures for mapping CNN features to MOS using these models, along with dataset splits and cross-validation, are detailed in this subsection.
4. *Discuss the trade-offs between accuracy and the cost, also whether other light weight models were checked.*
   - Added computational cost in **Table 7** (prediction speed, training time, model size) and use these to discuss the accuracy–efficiency trade-off. In addition to ResNet‑101 (PRIQA), several lighter backbones were evaluated, including ResNet‑18, DarkNet‑19, and EfficientNet‑B0. A short paragraph in **line 474 - 484** has been added adjacent to **Table 7** to make these trade-offs explicit and to recommend EfficientNet‑B0 and DarkNet‑53 for real‑time or resource‑constrained deployments, and ResNet‑101 when maximal accuracy is required.
5. *Clarify whether the model used were used with already trained weights and other parameters on your dataset.*
   - Clarified in the Deep Convolutional Neural Network (DCNN) subsection “Deep Convolutional Neural Network (DCNN)”, **lines 247–252**. All CNN backbones (EfficientNet-B0, DarkNet-53, Inception-ResNet-v2, DarkNet-19, ResNet-18, ResNet-50, ResNet-101, GoogleNet, and AlexNet) were initialized using ImageNet-pretrained weights provided by the MATLAB Deep Learning Toolbox, where the ImageNet dataset comprises over 1.2 million images across 1000 object categories. These pretrained models were used solely as feature extractors, and no additional fine-tuning of weights was performed on our dataset.
6. *Check all the grammatical, typographical and linguistic errors in manuscript and correct them in the revised submission.*
   - Reviewed the entire manuscript and corrected all grammatical, typographical, and linguistic errors. Improved sentence structures and overall readability to ensure the manuscript meets the required linguistic standards. These corrections have been applied consistently throughout the revised submission.

Thank you for your kind consideration of our work.

Yours Faithfully,

**NORRIMA MOKHTAR**

Senior Lecturer

Department of Electrical Engineering

Faculty of Engineering

University of Malaya

50603 Kuala Lumpur

Malaysia
