# Supplementary material for: Deep learning-based no-reference image quality assessment framework for Cryptosporidium spp. and Giardia spp
Source: PLoS One. 2026 Jan 20;21(1):e0341160. doi: 10.1371/journal.pone.0341160 (PMC12818675; doi:10.1371/journal.pone.0341160)
Supplement: Attachment — Submitted filename: Response_to_Reviewers_auresp_2.docx [file pone.0341160.s003.docx]

15 November 2025

Dear Editor and Reviewers,

Thank you very much for considering our manuscript entitled “**Deep Learning-Based No-Reference Image Quality Assessment Framework for Cryptosporidium spp. and Giardia spp.**”. We really appreciate your valuable suggestions and comments which help us to enhance the quality of our paper. We have revised the manuscript accordingly, with details below:

*Reviewers #2 comments:*

1. *The range of distortions considered by the authors’ are common, also the types are already defined on a small dataset, which does not justify the title as the size of dataset is not sufficient to train deep learning models. Thus, the manuscript makes it difficult to judge generalization capability of PRIQA model.*

- To better reflect the experimental setting, we now clearly present PRIQA as a domain-specific NR-IQA framework in a small-sample. This is reflected in the revised title and is also stated explicitly in the Conclusion, where we now describe the data as “synthetic distortions applied under controlled conditions in a small sample.” **(Refer line 678-680)**
- In the Introduction, we have added a paragraph highlighting recent NR-IQA and medical IQA studies that adopt a similar regression-based strategy on modest, domain-specific datasets **(Refer lines 130-138)**. For example, Sun et al. [22] trained a Random Forest regression model to map quantitative features from CT pulmonary angiography images to radiologists’ MOS on approximately 150 cases, and Hu et al. [23] used a Swin-Transformer-based regression head on the CSIQ database, which they explicitly describe as a small laboratory-synthesised dataset. These studies illustrate that regression from image features to subjective quality scores on carefully annotated but limited datasets is common practice in specialised imaging scenarios.
- In the Conclusion, we now briefly note that our design follows this regression-based NR-IQA pattern used in specialised domains **(Refer lines 687-690).**
- We have strengthened the limitations statement in the Conclusion to better reflect what is and is not claimed about generalization (**Refer line 680-682)**. Specifically, we state that the dataset consists of synthetic distortions applied under controlled conditions in a small sample, and that “the performance of the models on naturally distorted or clinically acquired images from different microscopes remains unexplored, which may affect external generalizability beyond the present controlled setting.” This makes clear that our results demonstrate good performance and internal generalization *within* the current controlled distortion setting, but as a general generalization to naturally distorted or multi-centre clinical data is not claimed and is identified as an important direction for future work.

2. *It has been observed that authors rely very much on ResNet-101, undoubtedly the performance of ResNet-101 is quite good with similar tested architectures, however, it is suggested to give a deeper analysis of learned features.*

- We have added a quantitative, layer-wise analysis of learned representations using linear CKA (Kornblith et al., 2019). Specifically, we now (i) introduce a new Methods subsection “Layer-wise CKA analysis” with citations **(lines 321–331)**, (ii) report depth-wise CKA for nine backbones across four distortions (JPEG, GWN, Speckle, SnP) in Fig. 5 **(lines 460–462)**, and (iii) list the exact probed layers for each backbone in Table 4 **(line 333)**. The analysis shows that ResNet-101 exhibits strong late-layer invariance near-identity for JPEG/Speckle (= 0.99–1.00) and robust to additive noise (GWN = 0.91, SnP = 0.97) while early layers are sensitive (e.g., GWN = 0.00, SnP = 0.04) with marked recovery at mid depth (e.g., SnP = 0.75, GWN = 0.64). We summarize these trends in Results subsection “Depth-wise robustness patterns” and explain why such late-layer stability is critical for microscopy (noise/illumination variability). Together, these additions provide the requested deeper feature analysis across architectures and offer a principled justification for selecting ResNet-101 as the PRIQA backbone.

3. *The manuscript shows the comparison with respect to 10 different NR-IQA algorithms, but the authors’ have not mentioned the basis of choosing them. Add a paragraph explaining the basic criteria to select these algorithms.*

- We added a paragraph in **Methods subsection NR-IQA (lines 374–378)** detailing our four selection criteria (citation impact, public code/weights, reproducibility on commodity hardware, and method diversity across NSS and deep CNNs) and enumerated the ten methods with citations.

4. *The discussion part could be strengthened if the authors’ add a clear description on how the models used are integrated into automated pipeline with all the operational constraints.*

- In Methods subsection DCNN, inference pipeline description has been added **(Refer line 256-268)**
- Implementation details have been added **(Refer line 269-274)**: Paragraph specifying hardware/software (MATLAB R2024a, Deep Learning Toolbox/Deep Network Designer, Windows 10, RTX 3060 Ti 8 GB, i5-10400, 16 GB RAM), precision (FP32), input-size policy, layer selection, feature dimension, model sizes, parameters, batch size, latency, throughput and peak VRAM.

5. *The authors have discussed the algorithm’s computational complexity for its implementation in real world applications but it still lacks clarity and could be further improved to assess the overall performance of the study.*

- **At lines 464-470:** Added a short **“Computational complexity and deployment implications”** paragraph linking **accuracy** to **cost** (Table 2), highlighting that **ResNet-101** provides the strongest accuracy at a manageable runtime, while **EfficientNet-B0** is preferred under tight throughput/VRAM budgets.

Thank you for your kind consideration of our work.

Yours Faithfully,

**NORRIMA MOKHTAR**

Senior Lecturer

Department of Electrical Engineering

Faculty of Engineering

University of Malaya

50603 Kuala Lumpur

Malaysia
