# Supplementary material for: Deep learning-based no-reference image quality assessment framework for Cryptosporidium spp. and Giardia spp
Source: PLoS One. 2026 Jan 20;21(1):e0341160. doi: 10.1371/journal.pone.0341160 (PMC12818675; doi:10.1371/journal.pone.0341160)
Supplement: Attachment — Submitted filename: Response_to_Reviewers_auresp_3.docx [file pone.0341160.s004.docx]

17^th^ December 2025

Dear Editor and Reviewers,

Thank you very much for considering our manuscript entitled “**Deep Learning-Based No-Reference Image Quality Assessment Framework for Cryptosporidium spp. and Giardia spp.**”. We really appreciate your valuable suggestions and comments which help us to enhance the quality of our paper. We have revised the manuscript accordingly, with details below:

*Reviewers #2 comments:*

1. *The integration of the models into automated pipeline including the constraints can be represented graphically or with a block diagram for clear and effortless understanding.*

- To improve clarity for clear and effortless understanding on the integration of models into automated pipeline. We have added a new figure to the revised manuscript. Figure 4 now presents the general experimental framework for CNN-based NR-IQA model selection, illustrating the end-to-end pipeline from image preprocessing and backbone feature extraction, through regression learning and validation strategies, to performance evaluation against ground-truth MOS. **Refer line (338 – 343).**

**Fig. 4 General framework for CNN-based NR-IQA model selection**

2. *The overall performance of the algorithm and the usefulness of your study still lack clarity. Elaborate.*

- We have clarified both the overall performance and the practical usefulness of the proposed method. In the last paragraph of the Introduction, we now explicitly state the practical role of PRIQA in real workflows. **Refer lines 187 – 194**.
- In the Conclusion section, we have expanded the discussion of how PRIQA can be integrated into parasitic microscopy workflows, particularly for public health inspections, environmental monitoring, and diagnostic systems in low-resource settings where microscopy remains the primary diagnostic tool. **Refer lines 705 – 710.**

3. *At some places, the vocabulary can be improvised as well as the linguistic, typographical mistakes should be checked and corrected.*

- To improves readability and flow, we have adjusted the order placements of subsections in Result & Discussion section. Spelling error as well as vocabulary has been improved in multiple sub sections. Refer “Manuscript with Changes” file to see the changes.

Thank you for your kind consideration of our work.

Yours Faithfully,

**NORRIMA MOKHTAR**

Senior Lecturer

Department of Electrical Engineering

Faculty of Engineering

University of Malaya

50603 Kuala Lumpur

Malaysia
